# Influence of multiple apolipoprotein A-I and B genetic variations on insulin resistance and metabolic syndrome in obstructive sleep apnea

**DOI:** 10.1186/s12986-020-00501-8

**Published:** 2020-09-29

**Authors:** Xinyi Li, Zhihui Fu, Huajun Xu, Jianyin Zou, Huaming Zhu, Zhiqiang Li, Kaiming Su, De Huai, Hongliang Yi, Jian Guan, Shankai Yin

**Affiliations:** 1 Department of Otorhinolaryngology-Head and Neck Surgery, Center of Sleep Medicine, Shanghai Jiao Tong University Affiliated Sixth People’s Hospital, 600 Yishan Road, Shanghai, 200233 People’s Republic of China; 2 Otolaryngological Institute of Shanghai Jiao Tong University, Yishan Road 600, Shanghai, 200233 People’s Republic of China; 3 Shanghai Key Laboratory of Sleep Disordered Breathing, Shanghai, People’s Republic of China; 4 Key Laboratory for the Genetics of Developmental and Neuropsychiatric Disorders, Bio-X Institutes, Ministry of Education, Shanghai Jiao Tong University, Huashan Road 1954, Shanghai, 200030 People’s Republic of China; 5 Department of Otorhinolaryngology, Huai’an Second People’s Hospital, Huai’an Hospital Affiliated to Xuzhou Medical University, 62 Huaihai South Road, Huai’an, 223002 Jiangsu People’s Republic of China

**Keywords:** Apolipoprotein A-I, Apolipoprotein B, Genetic risk score, Insulin resistance, Metabolic syndrome, Obstructive sleep apnea

## Abstract

**Background:**

The relationships between apolipoprotein A-I (APOA-I), apolipoprotein B (APOB) with insulin resistance, metabolic syndrome (MetS) are unclear in OSA. We aimed to evaluate whether the multiple single nucleotide polymorphism (SNP) variants of APOA-I and APOB exert a collaborative effect on insulin resistance and MetS in OSA.

**Methods:**

Initially, 12 APOA-I SNPs and 30 APOB SNPs in 5259 subjects were examined. After strict screening, four APOA-I SNPs and five APOB SNPs in 4007 participants were included. For each participant, the genetic risk score (GRS) was calculated based on the cumulative effect of multiple genetic variants of APOA-I and APOB. Logistic regression analyses were used to evaluate the relationships between APOA-I/APOB genetic polymorphisms, insulin resistance, and MetS in OSA.

**Results:**

Serum APOB levels increased the risk of insulin resistance and MetS adjusting for age, gender and BMI [odds ratio (OR = 3.168, *P* < 0.001; OR = 6.098, *P* < 0.001, respectively]. APOA-I GRS decreased the risk of insulin resistance and MetS after adjustments (OR = 0.917, *P* = 0.001; OR = 0.870, *P* < 0.001, respectively). APOB GRS had no association with insulin resistance (OR = 1.364, *P* = 0.610), and had weak association with MetS after adjustments (OR = 1.072, *P* = 0.042). In addition, individuals in the top quintile of the APOA-I genetic score distribution had a lower risk of insulin resistance and MetS after adjustments (OR = 0.761, *P* = 0.007; OR = 0.637, *P* < 0.001, respectively).

**Conclusions:**

In patients with OSA, cumulative effects of APOA-I genetic variations decreased the risk of insulin resistance and MetS, whereas multiple APOB genetic variations had no associations with insulin resistance and weak association with MetS.

## Background

Obstructive sleep apnea (OSA), characterized by upper airway obstruction during sleep resulting in breathing pauses, intermittent hypoxia, and fragmented sleep, affects 49.7% of men and 23.4% of women and is largely undiagnosed [1]. It is also commonly recognized as an important risk factor for insulin resistance and metabolic syndrome (MetS) [2–4].

Apolipoprotein A-I (APOA-I) and apolipoprotein B (APOB) are two main lipoproteins. APOA-I is a major apolipoprotein in high-density lipoprotein cholesterol (HDL-C) and manifests antiatherogenic properties [5]. APOB is present in very low-density lipoprotein (VLDL), intermediate-density lipoprotein, and low-density lipoprotein cholesterol (LDL-C) and may enhance atherothrombosis [5, 6]. Many clinical trials have revealed that APOA-I and APOB are independently associated with insulin resistance and MetS [7–9]. OSA is believed to be associated with APOA-I and APOB (i.e., in OSA, all sleep variables are positively correlated with the APOB/APOA-I ratio) [9]. Eight weeks of continuous positive airway pressure (CPAP) treatment can significantly decrease the APOB level [10]. Our previous study demonstrated that APOB/APOA-I increased the risk of insulin resistance, insulin resistance play a mediator between OSA and APOB/APOA-I [11]. However, whether APOA-I and APOB are independently associated with insulin resistance and MetS in OSA remains uncertain.

Both genetic and environmental factors play an important role in insulin resistance and MetS [12–15]. Although significant evidence links OSA to insulin resistance and MetS [3, 16], little is known about the roles of the genetic factors of lipoproteins involved in insulin resistance and MetS in OSA. Particularly, no current data on potential links between susceptibility genes for APOA-I and APOB and OSA-related insulin resistance and MetS are available.

Ordinarily, there is a tiny effect size of one single nucleotide polymorphism (SNP) to increase the risk of disease in a large number of variants. However, when the cumulative effect of a substantial fraction of variations reaches a certain threshold, the risk of disease is significantly increased [17]. Previous studies have used a cumulative effect model (genetic risk score, GRS) to identify risk factors of a certain disease. For example, total cholesterol (TC), total triglyceride (TG), HDL-C, and LDL-C genetic variants are associated with cardiovascular disease [18]; QT interval (measure of the time between the start of the Q wave and the end of the T wave in the heart's electrical cycle) duration genetic variants are associated with drug-induced QT prolongation [19]; and atrial fibrillation genetic variants are associated with future atrial fibrillation and stroke [20]. However, the relationships between the cumulative effects of multiple genetic variants of APOA-I, APOB, insulin resistance, and MetS in OSA remain unclear. In this study, we pooled multiple genetic variants of APOA-I and APOB to investigate the effects of APOA-I and APOB genotype on insulin resistance and MetS in the large-scale, clinical cohort study on OSA.

## Methods

### Subjects

Subjects who were initially suspected of having OSA were consecutively enrolled to participate in the ongoing Shanghai Sleep Heath Study (SSHS) (previously described in [21]). Subjects with non-OSA and moderate-to-severe OSA were chosen from the SSHS for an additional genomic study. Next, subjects that met the following inclusion and exclusion criteria were selected. Inclusion criteria were: older than 18 years of age without a return visit and previous treatment. Exclusion criteria were: (1) missing APOA-I and APOB data, (2) missing data on more than 15% of total SNPs, (3) regular use of lipid lowering drugs, (4) presence of a systemic disease (i.e., chronic pulmonary, renal, or hepatic failure), cancer, psychiatric disease, hyperparathyroidism, hypoparathyroidism, or polycystic ovarian syndrome; (5) other sleep disorders, such as restless leg syndrome or narcolepsy; (6) cardiovascular disease (i.e., angina, myocardial infarction, heart arrhythmia, or valvular heart disease); and (7) missing systolic blood pressure (SBP), TC, HDL-C, and fasting plasma glucose (FPG) data. Ultimately, 4007 participants were analyzed in this study that was approved by the Institutional Ethics Committee of Shanghai Jiao Tong University Affiliated Sixth People’s Hospital. Written informed consent was obtained from all subjects.

### Anthropometric and biochemical measurements

Waist circumference (WC) was measured at the middle of the lowest costal margin and iliac crest. Hip circumference (HC) was measured at the widest part of the buttocks. Neck circumference (NC) was measured at the level of the cricothyroid membrane. WC, HC, and NC were measured by trained investigators following standard protocols. Body mass index (BMI) was calculated as weight (in kilograms) divided by height squared (in meters). The waist-hip ratio (WHR) was calculated as WC divided by HC (in centimeters). SBP and diastolic blood pressure (DBP) were measured in triplicate after at least a 10-min rest using an automated electronic device (Omron Model HEM-752 Fuzzy, Omron Company), and the average value of the three readings was used for analysis.

A fasting blood sample was obtained the morning after polysomnographic monitoring. FPG, TC, TG, HDL-C, LDL-C, APOA-I, APOB, and apolipoprotein E were measured using an autoanalyzer (H-7600; Hitachi, Tokyo, Japan) in the hospital laboratory. Serum fasting insulin was measured using immunoassay. Homeostasis model assessment of insulin resistance (HOMA-IR) was calculated as fasting insulin (μIU/mL) × FPG (mmol/L)/22.5. HOMA-IR ≥ 2.5 was defined as insulin resistance [22]. Abnormal APOA-I and APOB were defined as serum levels < 1.20 and > 1.10 g/L, respectively, according to diagnostic criteria of the Joint Committee for Developing Chinese Guidelines on the Prevention and Treatment of Dyslipidemia in Adults [23]. A person had metabolic syndrome if presenting three or more of the following conditions [24]: (1) TG ≥ 150 mg/dL; (2) HDL-C < 40 mg/dL in men or < 50 mg/dL in women; (3) SBP ≥ 130 mmHg, DBP ≥ 85 mmHg, or diagnosed hypertension; (4) fasting glucose ≥ 100 mg/dL or drug treatment for type 2 diabetes; and (5) WC ≥ 90 cm in men or ≥ 80 cm in women.

### Polysomnographic evaluation and OSA definition

Overnight standard polysomnography (PSG, Alice 4 or 5; Respironics Inc., Pittsburgh, PA, USA) was used to obtain objective sleep parameters. An electroencephalogram, bilateral electroculogram, chin electromyogram, electrocardiogram, nasal and oral airflow, finger pulse oximetry, chest and abdominal movements, and body posture were recorded during sleep. Apnea was defined as cessation of airflow for ≥ 10 s, and hypopnea was defined as ≥ 50% reduction in airflow accompanied with ≥ 3% decrease in oxygen desaturation according to the 2007 American Academic Sleep Medicine criteria [25]. The severity of OSA was determined by the apnea–hypopnea index (AHI), and non-OSA, mild, moderate, and severe were defined as AHI < 5, 5–15, 15–30, and ≥ 30 per hour, respectively. The oxygen desaturation index was calculated as the number of episodes of oxygen desaturation ≥ 3% per hour during sleep. The micro-arousal index was calculated as the number of arousals per hour of sleep.

### SNP selection, genotyping and GRS calculation

We selected almost all of the reported APOA-I and APOB SNPs from large-scale genome-wide association studies and meta-analyses [26, 27], including 12 APOA SNPs (rs964184, rs9804646, rs12225230, rs11216162, rs5072, rs10047462, rs689243, rs10047459, rs888245, rs888246, rs625145, and rs12099358) and 30 APOB SNPs (rs11902417, rs1042034, rs1042031, rs2678379, rs1800479, rs676210, rs693, rs1041968, rs3749054, rs673548, rs2854725, rs12713956, rs12720828, rs10199768, rs12720838, rs679899, rs570877, rs520354, rs550619, rs597331, rs531819, rs1367117, rs1800481, rs934197, rs585967, rs7575840, rs563290, rs754524, rs754523, and rs562338). After filtering those variants in our genomic database, we excluded rs531819, rs1800481, and rs585967 because the call rates were < 95%. SNPs rs563290 and rs562338 were also excluded due to minor allele frequencies < 1%, which failed quality control. Finally, APOA-I SNPs rs964184, rs9804646, rs10047462, and rs888246, and APOB SNPs rs1042031, rs693, rs2854725, rs1367117, and rs12713956 met linkage disequilibrium (LD) < 0.2 and were analyzed in this study.

For GRS construction, we assumed an additive genetic model for each variant [28]. The weighted computation of APOA and APOB was calculated by multiplying each subject’s risk allele score (0, 1, or 2) by the SNP’s β coefficient from our data; values for each locus were then summed.

### Statistical analysis

Statistical analyses were performed using SPSS software (version 19.0, IBM Corp., Armonk, NY, USA). Continuous data are presented as the mean ± standard deviation (SD) for normalized variables and as the median (interquartile range) for skewed variables. Categorical variables are shown in proportions. The Hardy–Weinberg equilibrium test was performed for each variant before association analysis using PLINK (https://zzz.bwh.harvard.edu/plink/data.shtml). LD was performed at https://archive.broadinstitute.org/mpg/snap/ldsearchpw.php. Differences in baseline characteristics among groups were examined using the least-significant difference test, one-way analysis of variance, the chi-squared test, the independent samples *t*-test, or the Mann–Whitney *U* test according to the distribution characteristics of the data. Linear regressions were used to evaluate the associations between SNPs and serum APOA, APOB levels. We used logistic regression models to assess the OR of individuals in the top quintiles of the APOA and APOB GRS distributions with reference to individuals in the lowest quintile to examine the risk of moderate-to-severe OSA, insulin resistance, and MetS, both unadjusted and adjusted for age, gender, and BMI. Linear regression was used to evaluate the associations between GRS and clinical characteristics. Stepwise multivariate linear regression analysis was used to predict HOMA-IR. A two-tailed *P* value < 0.05 was considered statistically significant.

## Results

### Baseline characteristics

**Fig. 1:**
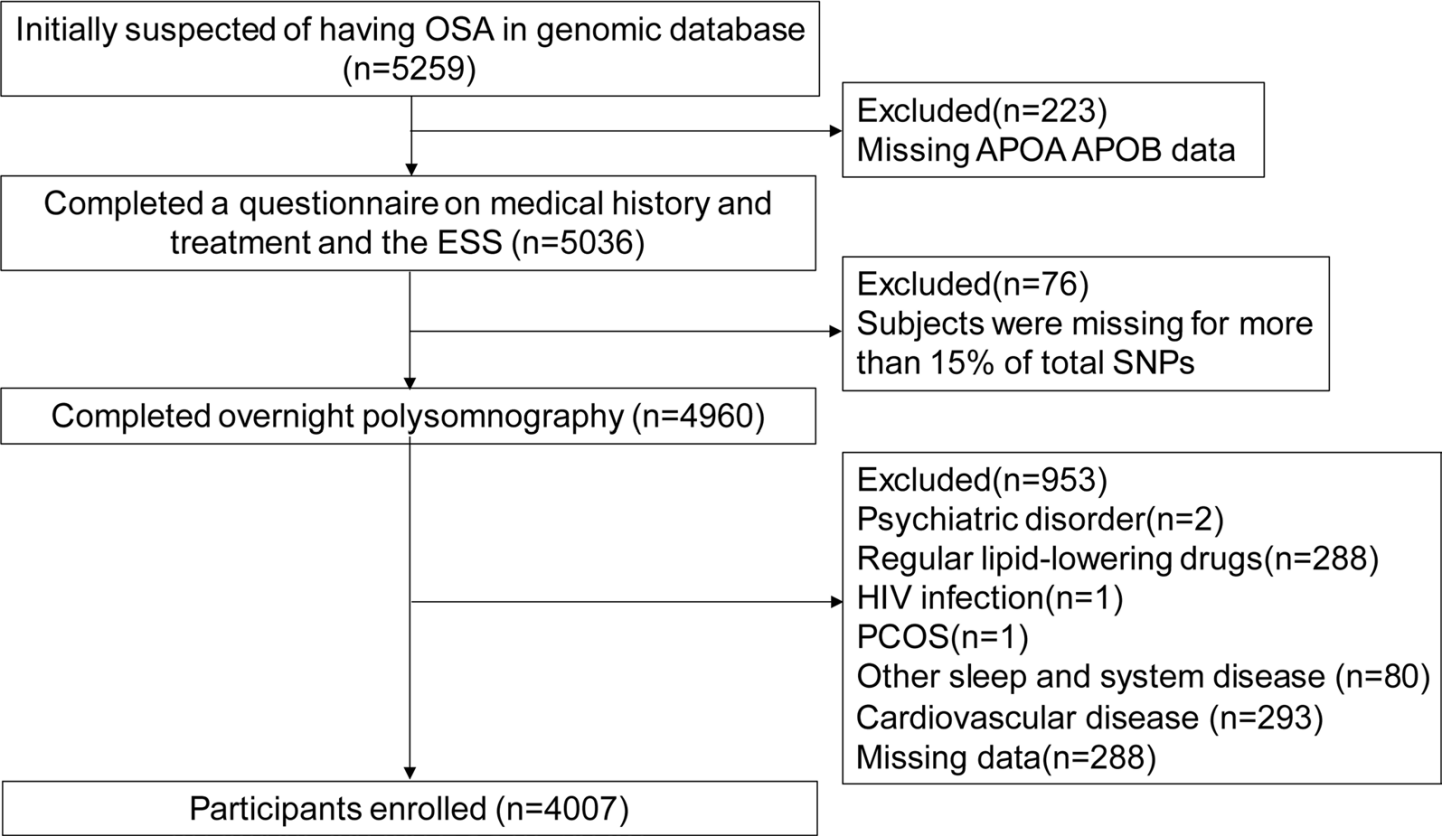
Enrollment flow chart for the study population. A total of 5259 patients in the genomic database of the Shanghai Sleep Health Study cohort were included. Ultimately, 4007 patients met the inclusion criteria and were enrolled in the current study

In total, 4007 eligible subjects (596 non-OSA, and 3411 moderate-to-severe OSA) were enrolled in this study (see flow chart in Fig. 1). Of the 4007 participants enrolled, 596 were non-OSA, 831 were moderate OSA and 2580 were severe OSA. Of the non-OSA subjects, the median age was 34 (range 29–43), the median HOMA-IR was 1.172 (range 1.11–2.55), median serum APOA-I was 1.092 (range 0.092–1.15) g/L, medium serum APOB was 0.77 (range 0.65–0.89) g/L, the percentage of insulin resistance was 26.8%, the percentage of metabolic syndrome was 27.2%, the median AHI value was 2 (range 0.8–3.4) events/h. Compared with non-OSA, patients with OSA were older and had higher serum concentrations of glucose, insulin, sleep parameters and ratio of smoking, drinking, prevalence of insulin resistance and percentage of MetS. OSA patients had higher levels of anthropometric parameter, such as SBP, DBP, BMI, NC, WC, HC, WHR. With the exception of serum APOA-I, all biochemistry parameters, demographic parameters and sleep parameters were also significantly different among the groups (Table 1). There were more subjects with insulin resistance and MetS in OSA group compared to non-OSA (*P* < 0.001). The percentages of insulin resistance in non-OSA, moderate, and severe OSA were 26.8%, 46.8%, and 63.4%, respectively. The percentages of MetS in non-OSA, moderate, and severe OSA were 27.2%, 50.2%, and 64.3%, respectively.

**Table 1:** Basic characteristics of the overall population by the severity of OSA

Variable	Non-OSA (n = 596)	Moderate OSA (n = 831)	Severe OSA (n = 2580)	*P* value
*Demographics*				
Age (years)	34 (29–43)	43 (35–53)	41 (34–51)	< 0.001
Male (%)	596 (100%)	675 (81.2%)	2332 (90.4%)	< 0.001
BMI (kg/m^2^)	24.2 (22.3–26.0)	26.0 (24.07–28.0)	27.7 (25.8–30.1)	< 0.001
NC (cm)	38 (36–40)	39 (37–41)	41 (39–43)	< 0.001
WC (cm)	89 (83–94)	94 (89–99)	99 (94–105)	< 0.001
HC (cm)	98 (94–101)	100 (96–104)	103 (99–108)	< 0.001
WHR	0.91 (0.87–0.95)	0.94 (0.91–0.98)	0.96 (0.93–1.0)	< 0.001
*Biochemistry assays*				
Glucose (mmol/L)	5.07 (4.71–5.36)	5.23 (4.9–5.69)	5.39 (5.05–5.95)	< 0.001
Insulin (uU/mL)	7.56 (5.2–11.34)	10.24 (7.04–14.48)	12.88 (8.92–18.96)	< 0.001
HOMA-IR	1.72 (1.11–2.55)	2.41 (1.56–3.48)	3.15 (2.08–4.84)	< 0.001
SBP (mmHg)	120 (112–128)	124 (115–134)	127 (120–138)	< 0.001
DBP (mmHg)	78 (71–82)	80 (71–87)	81 (76–90)	< 0.001
TC (mmol/L)	4.37 (3.84–4.96)	4.73 (4.17–5.40)	4.84 (4.28–5.44)	< 0.001
TG (mmol/L)	1.24 (0.84–1.84)	1.61 (1.14–2.29)	1.77 (1.26–2.61)	< 0.001
HDL-C (mmol/L)	1.03 (0.92–1.12)	1.02 (0.9–1.12)	1 (0.88–1.14)	< 0.001
LDL-C (mmol/L)	2.68 (2.25–3.18)	3 (2.51–3.51)	3.04 (2.53–3.55)	< 0.001
ApoA-I (g/L)	1.02 (0.92–1.15)	1.03 (0.92–1.17)	1.04 (0.93–1.16)	0.379
ApoB (g/L)	0.77 (0.65–0.89)	0.84 (0.73–0.98)	0.88 (0.76–1.0)	< 0.001
ApoB/ApoA-I	0.74 (0.61–0.89)	0.81 (0.67–0.97)	0.84 (0.71–0.98)	< 0.001
*Sleep apnea*				
AHI	2 (0.8–3.4)	21.8 (18.3–25.7)	58.2 (45.1–70.5)	< 0.001
Minimum SaO_2_	92 (89–95)	83 (77–87)	71 (62–79)	< 0.001
ODI	2.1 (0.9–3.8)	22.2 (17.3–28.0)	57.9 (44–72.2)	< 0.001
MAI	14.4 (10.3–22.5)	22.2 (14.2–31.6)	36.7 (21.6–56.4)	< 0.001
*Medical history*				
ESS	7 (3–10)	7 (4–11)	11 (7–15)	< 0.001
Non-smoker, N (%)	372 (62.4%)	488 (58.7%)	1300 (50.4%)	< 0.001
Non-drinker, N (%)	284 (47.7%)	405 (48.7%)	1131 (43.8%)	0.024
IR (%)	160 (26.8%)	389 (46.8%)	1636 (63.4%)	< 0.001
Met S (%)	162 (27.2%)	417 (50.2%)	1660 (64.3%)	< 0.001

The data are presented as means and standard deviation; skewed data are presented as the median (IQR), and categorical data as the number (percentage)

*GRS* genetic risk score, *BMI* body mass index, *NC* neck circumference, *WC* waist circumference, *HC* hip circumference, *WHR* waist/hip ratio, *HOMA-IR* homeostasis model assessment for insulin resistance, *SBP* systolic blood pressure, *DBP* diastolic blood pressure, *TC* total cholesterol, *TG* triglyceride, *HDL-C* high-density lipoprotein cholesterol, *LDL-C* low-density lipoprotein; cholesterol; *ApoA-I* apolipoprotein A-I, *ApoB* apolipoprotein B, *ESS* Epworth Sleepiness Scale, *AHI* apnoea–hypopnea index, *SaO2* oxygen saturation, *ODI* oxygen desaturation index, *MAI* micro-arousal index

The basic characteristics of the SNPs and SNP’s β coefficients in GRS construction are listed in Table 2. APOA-I SNP rs964184, rs10047462, rs888246 had negative correlations (ß = − 0.013, − 0.008, − 0.014, respectively) while rs9804646 had positive correlation with serum APOA-I levels (ß = 0.015). APOB SNP 1042031, rs693, rs1367117 associated with APOB levels positively (ß = 0.017, 0.009, 0.012, respectively), while rs2854725, rs12713956 associated with APOB levels negatively (both ß = − 0.03).

**Table 2:** Information of each individual SNP

SNP	Gene	Chromosome	Position	Minor allele	Major allele	Risk allele	Minor allele frequency	ß	P*
rs964184	APOA-I	11	116648917	G	C	C	0.216	− 0.013	0.012
rs9804646	APOA-I	11	116665079	T	C	T	0.216	0.015	0.006
rs10047462	APOA-I	11	116722041	G	T	T	0.484	− 0.008	0.076
rs888246	APOA-I	11	116724232	T	C	C	0.0052	− 0.014	0.16
rs1042031	APOB	2	21225753	T	C	T	0.044	0.017	0.11
rs693	APOB	2	21232195	A	G	G	0.049	0.009	0.37
rs2854725	APOB	2	21237786	G	T	T	0.13	− 0.03	< 0.001
rs1367117	APOB	2	21263900	A	G	G	0.12	0.012	0.008
rs12713956	APOB	2	21241505	G	A	G	0.042	− 0.03	0.004

*APOA-I* Apolipoprotein A-I, *APOB* Apolipoprotein B

P* value was adjusted for age, gender, BMI as confounding factors

We also assessed the clinical characteristics of the participants in the top quintiles of the APOA GRS and APOB GRS compared with those in the bottom fifth quintiles (Additional file 1: Tables S1 and S2, respectively). The basic characteristics of insulin, insulin resistance, and TG were lower in the highest quintile of APOA GRS than in the lowest quintile of APOA GRS, whereas HDL-C, LDL-C, APOA, and APOA/APOB were higher (all *P* < 0.05; Additional file 1: Table S1). As expected, TC, APOB, and LDL-C levels were higher in the highest quintile of APOB GRS than in the lowest quintile of APOB GRS (all *P* < 0.05, Additional file 1: Table S2).

### Associations between common APOA-I and APOB genotypes and serum APOA-I and APOB levels

All SNPs of APOA-I and APOB were in Hardy–Weinberg equilibrium (*P* > 0.05). The associations of SNPs with serum APOA-I and APOB levels are summarized in Table 2. APOA-I rs964184 and rs9804646 were associated with APOA-I (β = − 0.013, *P* = 0.021; β = 0.015, *P* = 0.006). APOB SNP rs2854725 was significantly associated with APOB (β = − 0.03, *P* < 0.001). APOB SNPs rs1367117 and rs12713956 were moderately associated with increased APOB levels (β = 0.012, *P* = 0.008; β = − 0.03, *P* = 0.004, respectively) (Table 2).

### Associations of serum APOA-I and APOB and their GRS with insulin resistance and MetS risks

The associations between each SNP of APOA-I and APOB with insulin resistance and MetS are listed in Additional file 1: Table S3. APOA-I SNPs rs9804646 and rs888246 were associated with insulin resistance (OR = 0.856, 95% confidence interval [CI] 0.756–0.968, *P* = 0.013; OR = 1.340, 95% CI 1.069–1.680, *P* = 0.011) after adjustment. APOA-I SNPs rs964184, rs9804646, and rs888246 were significantly associated with MetS (OR = 1.353, 95% CI 1.201–1.523, *P* < 0.01; OR = 0.777, 95% CI 0.69–0.874, *P* < 0.01; OR = 1.274, 95% CI 1.024–1.586, *P* = 0.03, respectively) after adjustment. For APOB, only rs2854725 was associated with MetS (OR = 0.829, 95% CI 0.718–0.956, *P* = 0.01) after adjusting for age, gender, and BMI.

Serum APOA-I levels decreased the risk of insulin resistance and MetS (Table 3) (OR = 0.573. *P* < 0.001; OR = 0.131, *P* < 0.001), and had no relationship with insulin resistance after adjustment (OR = 0.8233, *P* = 0.308). Serum APOA-I levels remained to decrease the risk of MetS after adjustment (OR = 0.09, *P* < 0.001). Serum APOB levels increased the risk of insulin resistance and MetS (OR = 0.573, *P* < 0.001; OR = 0.131, *P* < 0.001), which remained after adjusting for age, gender, and BMI (OR = 3.168, *P* < 0.001; OR = 6.098, *P* < 0.001). APOA-I GRS decreased insulin resistance (OR = 0.923, 95% CI 0.880–0.968, *P* < 0.001) and MetS (OR = 0.886, 95% CI 0.845–0.929, *P* < 0.001) significantly, which remained after adjusting for age, gender, and BMI (all *P* < 0.001). APOB GRS was not associated with insulin resistance and MetS (*P* > 0.05). Subjects with OSA had higher APOB/APOA-I and APOB levels (OR = 7.610, 95% CI 4.886–11.852, *P* < 0.001; OR = 31.683, 95% CI 18.231–55.059, *P* < 0.001, respectively), even after adjusting for age, gender, and BMI.

**Table 3:** Association of risk of APOA-I, APOB level and their GRS with risks of insulin resistance and MetS

	Non-HOMA-IR vs HOMA-IR			Non-MetS vs MetS			Non-OSA vs moderate to severe OSA		
	OR	95%CI	*P*	OR	95%CI	*P*	OR	95%CI	*P*
APOA-I GRS	0.923	0.880–0.968	< 0.001	0.886	0.845–0.929	< 0.001	0.983	0.920–1.049	0.600
APOA-I GRS*	0.917	0.869–0.967	0.001	0.870	0.827–0.916	< 0.001	0.994	0.924–1.070	0.874
APOB GRS	1.011	0.950–1.076	0.734	1.060	0.997–1.128	0.063	1.011	0.927–1.101	0.811
APOB GRS*	1.364	1.330–1.339	0.610	1.072	1.003–1.147	0.042	1.000	0.907–1.104	0.993
APOB/APOA-I	4.551	3.379–6.129	< 0.001	19.021	13.629–26.545	< 0.001	7.610	4.886–11.852	< 0.001
APOB/APOA-I *	2.285	1.657–3.150	< 0.001	14.488	10.093–20.797	< 0.001	3.237	1.993–5.258	< 0.001
APOA-I	0.573	0.414–0.792	< 0.001	0.131	0.093–0.184	< 0.001	1.307	0.828–2.063	0.250
APOA-I *	0.823	0.566–1.196	0.308	0.09	0.061–0.132	< 0.001	1.336	0.788–2.267	0.282
APOB	6.677	4.654–9.578	< 0.001	12.095	8.349–17.522	< 0.001	31.683	18.231–55.059	< 0.001
APOB*	3.168	2.139–4.691	< 0.001	6.098	4.109–9.051	< 0.001	8.582	4.653–45.830	< 0.001

*HOMA-IR* homeostasis model assessment of insulin resistance, *GRS* genetic risk score, *Met S* metabolic syndrome, *APOA-I* Apolipoprotein A-I, *APOB* Apolipoprotein B

^*^Adjust for age, gender, BMI

We also stratified APOA-I and APOB GRS into quintiles. When compared with the bottom quintile, subjects in the top quintile of the APOA-I GRS group had a lower risk of insulin resistance and MetS (Table 4) [OR = 0.753 (0.63–0.90), *P* = 0.002; OR = 0.651 (0.546–0.777), *P* < 0.001], even after adjusting for age, gender, and BMI [OR = 0.761 (0.623–0.929), *P* = 0.007; OR = 0.637 (0.526–0.773), *P* < 0.001]. APOB GRS was not associated with insulin resistance (OR = 1.364, *P* = 0.610), and had weak associations with MetS (OR = 1.072, *P* = 0.042). Linear regression analysis revealed that APOA-I GRS was associated with decreased insulin, TG, HOMA-IR, APOB/APOA-I, and elevated HDL-C, LDL-C, and APOA-I levels (all *P* < 0.05, Additional file 1: Table S4), even after adjustment. APOB GRS was associated with elevated TC, LDL-C, APOB, and APOB/APOA-I levels (all *P* < 0.001, Additional file 1: Table S5).

**Table 4:** Risk of OSA, insulin resistance and MetS according to quintile of APOA-I GRS and APOB GRS

	Top vs bottom quintile unadjusted analysis			Top vs bottom quintile adjusted analysis*		
	OR	95%CI	*P*	OR*	95% CI*	P*
*APOA-I GRS*						
Non-OSA vs moderate to severe OSA	0.812	0.635–1.039	0.098	0.855	0.654–1.118	0.252
Non-insulin resistance vs insulin resistance	0.753	0.630–0.899	*0.002*	0.761	0.623–0.929	*0.007*
Non-MetS vs MetS	0.651	0.546–0.777	*< 0.001*	0.637	0.526–0.773	*< 0.001*
*APOB GRS*						
Non-OSA vs moderate to severe OSA	0.997	0.768–1.295	0.984	0.98	0.738–1.301	0.889
Non-insulin resistance vs insulin resistance	1.019	0.848–1.225	0.839	1.032	0.841–1.267	0.761
Non-MetS vs MetS	1.154	0.962–1.384	0.123	1.182	0.971–1.439	0.096

*OSA* obstructive sleep apnea, *HOMA-IR* homeostasis model assessment of insulin resistance, *GRS* genetic risk score, *MetS* metabolic syndrome, *AHI* apnoea–hypopnea index, *APOA-I* Apolipoprotein A-I, *APOB* Apolipoprotein B

^*^Adjust for age, gender, BMI

### Percentages of independent contributors of HOMA-IR

To reveal the percentages of independent contributors of HOMA-IR, stepwise multivariate linear regression analysis was performed. APOA-I GRS, age, gender, and BMI were included in model 1. APOA GRS, gender, and BMI explained 0.099%, 0.14%, and 18% of HOMA-IR, respectively (Additional file 1: Table S6). As AHI was identified as an important marker of OSA, we included AHI in model 2, APOA-I GRS, gender, BMI, and AHI explained 0.1%, 0.14%, 18%, and 0.94% of HOMA-IR, respectively (all *P* < 0.05, Additional file 1: Table S6).

## Discussion

Our study was the first to comprehensively examine the roles of APOA-I and APOB levels and their genetic variations on insulin resistance, MetS, and OSA using current large-scale sampling and strict data acquisition. OSA Patients had higher TC, TG, LDL-C APOB levels and APOB/APOA-I ratios than those without OSA. Subjects with OSA were more obese and had higher levels of glucose, SBP, DBP, and insulin resistance than those without OSA. Not only did serum APOA-I and APOB levels correlate with insulin resistance and MetS, but cumulative genetic variants of APOA-I and APOB also exhibited effects on insulin resistance and MetS in OSA. Individuals in the top quintile of APOA-I genetic score distributions tended to have a lower risk of insulin resistance and MetS.

Obesity is one of the most important risk factor of OSA, and the major contributing factor to the development of insulin resistance and MetS. Thus it is most likely that OSA and insulin resistance are parallel outcomes of obesity while they might not have a direct causality relationship with each other. For the past years, various experiments have been able to better unravel complex mechanisms via in-vitro and animal models, prospective observational and treatment studies revealed that the association between metabolic disease (including insulin resistance, obesity, metabolic syndrome) and OSA is bi-directional and feedforward [4, 29]. The interrelationships among OSA, insulin resistance, MetS, dyslipidemia, obesity was multifaceted and complicated. The manuscript aimed to evaluate the influence of lipids multiple genetic variants (APOA and APOB) on insulin resistance and MetS in OSA patients using obesity as a confounding factor.

It has reported that lower APOA-I level was associated with insulin resistance in patients with impaired glucose tolerance [7] and a higher prevalence of MetS [30]. The APOB level predicted the incidence of MetS in a 5-year follow-up study [8]. The relationship between APOA-I, APOB level and metabolic disease in OSA had been rarely studied. Our study suggests serum APOA-I, APOB level associated insulin resistance and MetS in OSA, and APOA-I and APOB were involved in metabolism and probably further increase cardiovascular disease risk.

APOA-I and APOB levels are not only influenced by environmental factors, such as diet and exercise, but are also subject to genetic regulation [31, 32]. Thus, APOA-I and APOB genetic variations may have a causal effect on insulin resistance and MetS. Previous studies have focused on the relationship between APOA-I and APOB genetic variations and serum lipid traits [31, 33, 34]. It had been reported that there was an ethnic and gender specific association between the APOA rs964184 with lower HDL-C, higher TG and lowers APOA levels in Chinese populations [31]. The sample was small, and the results were lack of the consideration of the impact of other SNP and diseases on serum lipids levels. APOA rs964184 was also associated with serum TG levels [35, 36], metabolic syndrome [35], coronary heart disease [37], and hemorrhagic stroke risk [38]. The APOA rs964184 study on OSA and insulin resistance has not been reported yet. Our results revealed that rs964184 had negative associations with serum APOA levels but had no associations with insulin resistance in a large cross-sectional study. APOB rs1042031(EcoRI) has been widely used to study coronary heart disease (CHD) [39] and Steroid-Induced Osteonecrosis of the femoral head (SONFH) [40]. They found APOB rs1042031 confers a moderate risk for CHD [39] and increase the SONFH risk with moderate levels of evidence. APOB rs693 [41], rs2854725 [42] and rs1367117 [43, 44] were associated with serum APOB levels, further associated with familial hypercholesterolemia [45] and heart-related traits [46], predicted the risk of CHD [42], maternally-derived effect on BMI [43].There were fewer studies about APOA SNP rs9804646, rs10047462, rs888246 and APOB rs12713956. Data on APOA-I and APOB genetic polymorphisms in insulin resistance and MetS are still lacking. Until now, the effects of multiple genetic variants about APOA and APOB have not been studied yet, especially in a specific disease population. Our data indicate that genetic variants of APOA-I and APOB SNPs play different roles in metabolic disorders, such as APOA-I rs9804646 decreased the risk of insulin resistance and MetS, but APOA-I rs888246 increased the risk of insulin resistance and MetS. There is a tiny effect of one SNP for the disease development. Thus, we use the GRS model to study the effect of multiple genetic variations.

The GRS is a convenient way to summarize a number of genetic variants associated with an individual’s genotype. The GRS does not change over time and holds the advantage that it can be used to assess the risk of metabolic dysfunction at any age from birth on. The GRS is always used in Mendelian randomization analysis to estimate the causal effect of a risk factor on an outcome [47]. This facilitates the use of genetic information, either alone or in combination, with other factors in clinical and research settings. Prospective studies have used the GRS to assess the cumulative effects of TC, TG, HDL-C and LDL-C related genetic variations on blood lipid levels, coronary events, and cardiovascular disease [18]. With the increasing availability of multiple genetic variants associated with lipids, it is becoming increasingly common to study associations with allele scores.

Our study was the first to screen 42 genetic variants and ultimately combine four APOA-I SNPs and five APOB SNPs (using the GRS model) to comprehensively examine the genetic roles of APOA-I and APOB in insulin resistance and MetS in OSA. In our study cumulative APOA-I genetic variations (APOA-I GRS) decreased the risk of insulin resistance and MetS, whereas cumulative APOB genetic variations (APOB GRS) increased the risk of MetS in OSA. The APOA-I gene is believed to be stimulated by insulin through SP-1 binding elements [48], and genetic variations of APOA-I may affect the binding site. Gene-diet interactions may also contribute to MetS [49, 50]. Because APOB SNPs are related to lipids [51, 52], the GRS of APOB may be beyond the interval of the association with insulin resistance. Future clinical trials as well as rodent studies should be designed to explore the potential mechanisms involved.

Both genetic and environmental factors are important contributors to insulin resistance. Our data indicate that environmental factors, such as BMI and AHI contribute more to HOMA-IR than do genetic variations. Genetic and environmental correlations for the same disease are complex [53]. Risk factors of OSA include obesity, age, male sex, and genetic background [54], and obesity is considered the most important risk factor [54, 55]. Therefore, in OSA-related insulin resistance, there should be more emphasis on environmental interventions than on genetic breakthroughs.

Our study aimed to obtain high quality results by using a large sample size, laboratory-based PSG, unified serological examination, and standard questionnaires. In addition, we used multiple SNPs in a GRS model to evaluate the prediction of individual risk for metabolic disorders. However, several limitations of the present study should be noted. First, although we aimed to collect a sufficient amount of SNPs, several APOA-I and APOB SNPs may have been omitted. Furthermore, more complex genetic variants, including indels and structural variants, were not considered. The effects of SNP–SNP and gene-environment interactions were not modeled. Second, although we made efforts to minimize limitations by building our large sample population using subjects with relatively homogeneous lifestyles and ethnicity and adjusted for common confounding factors, such as age, sex, and BMI, but other more sophisticated environmental factors, such as economic status, exercise, and lifestyle, were not considered in this study. Third, we quantified the effects of multiple genetic variations, further studies on the mechanisms are still needed. The study was cross-sectional rather than prospective and community-based design, and could not provide the causative evidence. Future clinical trials, as well as animal studies of genetics-derived OSA, will better shed light on these complex mechanisms.

## Conclusions

In conclusion, both the protective effects of multiple APOA-I genetic variants and damaging effects of APOB genetic variations impact the biomarkers of OSA patients. Obviously, the different cumulative effects of genes increase the complexity of metabolic disorders in OSA.

## Supplementary information

**Additional file 1: Table S1.** Basic characteristics of the top vs bottom quintile APOA GRS; **Table S2.** Basic characteristics of the top vs bottom quintile APOB GRS; **Table S3.** The associations between SNPs with insulin resistance and MetS. **Table S4.** Linear regression of APOA GRS with clinical characteristics; **Table S5.** Linear regression of APOB GRS with clinical characteristics; **Table S6.** The stepwise multivariate linear regression model for predicting HOMA-IR.

## Data Availability

All data generated or analyzed during this study can provide if needed.

## Abbreviations

APOA-I: Apolipoprotein A-I
APOB: Apolipoprotein B
MetS: Metabolic syndrome
OSA: Obstructive sleep apnea
SNP: Single nucleotide polymorphism
GRS: Genetic risk score
LD: Linkage disequilibrium
HOMA-IR: Homeostasis model assessment of insulin resistance
BMI: Body mass index
TC: Total cholesterol
TG: Triglycerides
HDL-C: High-density lipoprotein cholesterol
LDL-C: Low-density lipoprotein cholesterol
