# Supplementary material for: Influence of multiple apolipoprotein A-I and B genetic variations on insulin resistance and metabolic syndrome in obstructive sleep apnea
Source: Nutr Metab (Lond). 2020 Sep 29;17:83. doi: 10.1186/s12986-020-00501-8 (PMC7523361; doi:10.1186/s12986-020-00501-8)
Supplement: Supplementary file 1 — Additional file 1: Table S1. Basic characteristics of the top vs bottom quintile APOA GRS; Table S2. Basic characteristics of the top vs bottom quintile APOB GRS; Table S3. The associations between SNPs with insulin resistance and MetS. Table S4. Linear regression of APOA GRS with clinical characteristics; Table S5. Linear regression of APOB GRS with clinical characteristics; Table S6. The stepwise multivariate linear regression model for predicting HOMA-IR. [file 12986_2020_501_MOESM1_ESM.docx]

Table S1. Basic characteristics of the top vs bottom quintile APOA GRS

| Characteristics | Bottom quintile | Top quintile | P |
| --- | --- | --- | --- |
| N | 1011 | 1014 |  |
| **Demographics** |  |  |  |
| Age, years | 40(33-50) | 41(33-52) | 0.607 |
| Male (%) | 905(89.5%) | 906(89.3%) | 0.942 |
| BMI, Kg/m2 | 26.83(24.68-29.39) | 26.67(24.44-29.3) | 0.042 |
| NC, cm | 40(38-42) | 40(38-42) | 0.09 |
| WC, cm | 97(91-103) | 96(90-103) | 0.145 |
| HC, cm | 101(97-106) | 101(97-106) | 0.065 |
| WHR | 0.95(0.92-0.99) | 0.95(0.92-0.99) | 0.579 |
| **Biochemistry assays** |  |  |  |
| Glucose (mmol/L) | 5.34(4.99-5.87) | 5.27(4.92-5.78) | 0.381 |
| Insulin (uU/ml) | 12.0(8.0-17.6) | 10.98(7.24-16.87) | **0.005** |
| HOMA-IR | 2.9(1.85-4.52) | 2.63(1.66-4.19) | **0.014** |
| SBP, mmHg | 125(118-135) | 125(117-136) | 0.333 |
| DBP, mmHg | 80(75-89) | 80(74-89) | 0.862 |
| TC | 4.73(4.2-5.35) | 4.68(4.11-5.34) | 0.12 |
| TG | 1.83(1.27-2.74) | 1.50(1.08-2.10) | **<0.001** |
| HDL-C | 0.98(0.87-1.12) | 1.03(0.91-1.19) | **<0.001** |
| LDL-C | 2.92(2.41-3.40) | 3.01(2.50-3.55) | **0.001** |
| APOA-I | 1.02(0.92-1.15) | 1.05(0.93-1.18) | **0.002** |
| APOB | 0.85(0.75-0.97) | 0.84(0.73-0.97) | 0.082 |
| APOA-I/APOB | 1.21(1.02-1.44) | 1.26(1.06-1.49) | **0.001** |
| **Sleep apnea** |  |  |  |
| AHI | 44(21.8-64) | 41.8(20.3-62.0) | 0.107 |
| Minimum SaO2 | 77(67-85) | 77(67-86) | 0.449 |
| ODI | 43.1(21.6-65.5) | 42.1(19.9-62.8) | 0.222 |
| MAI | 29.3(16.9-50.0) | 27.4(16.1-46.4) | 0.051 |
| **Medical history** |  |  |  |
| ESS | 9(5-14) | 9(6-13) | 0.844 |
| Non-smoker, N(%) | 533(52.7%) | 540(53.3%) | 0.824 |
| Non-drinker, N(%) | 446(44.1%) | 487(48.0%) | 0.082 |
| IR (%) | 595(58.9%) | 523(51.6%) | 0.002 |
| Met S (%) | 616(60.9%) | 511(50.4%) | <0.001 |

The data are presented as means and standard deviation; skewed data are presented as the median (IQR), and categorical data as the number (percentage). Differences in the baseline characteristics among the four groups were examined using the polynomial linear trend test for continuous variables and the linear-by-linear association test for dichotomous variables. GRS: genetic risk score; BMI, body mass index; NC, neck circumference; WC, waist circumference; HC, hip circumference; WHR, waist/hip ratio; HOMA-IR, homeostasis model assessment for insulin resistance; SBP, systolic blood pressure; DBP, diastolic blood pressure; TC, total cholesterol; TG, triglyceride; HDL-C, high-density lipoprotein cholesterol; LDL-C, low-density lipoprotein; cholesterol; APOA-I, apolipoprotein A-I; APOB, apolipoprotein B; ESS, Epworth Sleepiness Scale; AHI, apnoea–hypopnea index; SaO2, oxygen saturation; ODI, oxygen desaturation index; MAI, micro-arousal index; MetS, metabolic syndrome.

Table S2. Basic characteristics of the top vs bottom quintile APOB GRS

| Characteristics | Bottom quintile | Top quintile | P |
| --- | --- | --- | --- |
| N | 936 | 941 |  |
| **Demographics** |  |  |  |
| Age, years | 40(33-50) | 41(34-50) | 0.644 |
| Male (%) | 844(90.2%) | 837(88.9%) | 0.407 |
| BMI, Kg/m2 | 26.57(24.68-29.36) | 26.64(24.46-29.32) | 0.722 |
| NC, cm | 40(38-42) | 40(38-42) | 0.266 |
| WC, cm | 97(90-103) | 97(91-103) | 0.217 |
| HC, cm | 102(97-106) | 101(97-106) | 0.412 |
| WHR | 0.95(0.91-0.99) | 0.95(0.92-0.99) | 0.286 |
| **Biochemistry assays** |  |  |  |
| Glucose (mmol/L) | 5.29(4.9-5.77) | 5.27(4.94-5.71) | 0.561 |
| Insulin (uU/ml) | 11.29(7.52-16.31) | 2.73(1.75-2.73) | 0.611 |
| HOMA-IR | 2.70(1.72-4.19) | 125(117-136) | 0.648 |
| SBP, mmHg | 125(117-135) | 125(117-136) | 0.994 |
| DBP, mmHg | 80(74-88) | 80(75-89) | 0.559 |
| TC | 4.64(4.09-5.26) | 4.83(4.19-5.45) | **0.002** |
| TG | 1.58(1.12-2.78) | 1.71(1.19-2.47) | 0.541 |
| HDL-C | 1.02(0.89-1.17) | 1.01(0.90-1.14) | 0.785 |
| LDL-C | 2.93(2.40-3.38) | 3.04(2.52-3.59) | **<0.001** |
| APOA-I | 1.04(0.93-1.17) | 1.04(0.94-1.16) | 0.93 |
| APOB | 0.83(0.72-0.95) | 0.87(0.76-1.00) | **<0.001** |
| APOA-I/APOB | 1.26(1.06-1.5) | 1.19(1.02-1.44) | 0.001 |
| **Sleep apnea** |  |  |  |
| AHI | 43.6(21.9-63.1) | 46.8(23.2-63.3) | 0.293 |
| Minimum SaO2 | 77(67-85) | 77(67-85) | 0.55 |
| ODI | 43.6(21.5-64.9) | 47.2(22.9-64.5) | 0.558 |
| MAI | 29.2(16.8-48.5) | 28.4(16.7-48.3) | 0.781 |
| **Medical history** |  |  |  |
| ESS | 9(6-14) | 9(6-14) | 0.899 |
| Non-smoker, N(%) | 505(54.0%) | 514(54.6%) | 0.781 |
| Non-drinker, N(%) | 423(45.2%) | 436(46.3%) | 0.643 |
| IR (%) | 509(54.4%) | 515(54.7%) | 0.839 |
| Met S (%) | 496(53.0%) | 532(56.5%) | 0.123 |

The data are presented as means and standard deviation; skewed data are presented as the median (IQR), and categorical data as the number (percentage). Differences in the baseline characteristics among the four groups were examined using the polynomial linear trend test for continuous variables and the linear-by-linear association test for dichotomous variables. GRS: genetic risk score; BMI, body mass index; NC, neck circumference; WC, waist circumference; HC, hip circumference; WHR, waist/hip ratio; HOMA-IR, homeostasis model assessment for insulin resistance; SBP, systolic blood pressure; DBP, diastolic blood pressure; TC, total cholesterol; TG, triglyceride; HDL-C, high-density lipoprotein cholesterol; LDL-C, low-density lipoprotein; cholesterol; APOA-I, apolipoprotein A-I; APOB, apolipoprotein B; ESS, Epworth Sleepiness Scale; AHI, apnoea–hypopnea index; SaO2, oxygen saturation; ODI, oxygen desaturation index; MAI, micro-arousal index; MetS, metabolic syndrome.

Table S3. The associations between SNPs with insulin resistance and MetS.

|  |  | non-HOMA-IR vs HOMA-IR | | | |  | non-MetS vs MetS | | | |
| --- | --- | --- | --- | --- | --- | --- | --- | --- | --- | --- |
|  |  | OR (95%CI) | P | OR (95%CI)* | P* |  | OR (95%CI) | P | OR (95%CI)* | P* |
| APOA-I | rs964184 | 1.084(0.973-1.208) | 0.142 | 1.117(0.989-1.261) | 0.074 |  | 1.286(1.154-1.433) | **<0.001** | 1.353(1.201-1.523) | **<0.001** |
|  | rs9804646 | 0.860(0.771-0.960) | **0.007** | 0.856(0.756-0.968) | **0.013** |  | 0.795(0.713-0.886) | **<0.001** | 0.777(0.69-0.874) | **<0.001** |
|  | rs10047462 | 1.053(0.963-1.151) | 0.259 | 1.045(0.946-1.155) | 0.386 |  | 1.032(0.945-1.127) | 0.482 | 1.043(0.947-1.149) | 0.39 |
|  | rs888246 | 1.373(1.119-1.684) | **0.002** | 1.340(1.069-1.680) | **0.011** |  | 1.271(1.039-1.55) | **0.02** | 1.274(1.024-1.586) | **0.03** |
|  |  |  |  |  |  |  |  |  |  |  |
| APOB | rs1042031 | 1.109(0.891-1.382) | 0.354 | 1.127(0.880-1.443) | 0.345 |  | 1.162(0.933-1.447) | 0.181 | 1.193(0.938-1.516) | 0.151 |
|  | rs693 | 1.048(0.851-1.291) | 0.659 | 0.969(0.767-1.224) | 0.792 |  | 1.023(0.833-1.256) | 0.827 | 0.945(0.756-1.181) | 0.618 |
|  | rs2854725 | 0.986(0.864-1.126) | 0.838 | 0.988(0.852-1.147) | 0.878 |  | 0.861(0.756-0.981) | **0.025** | 0.829(0.718-0.956) | **0.01** |
|  | rs1367117 | 0.974(0.849-1.118) | 0.712 | 0.952(0.814-1.112) | 0.533 |  | 0.925(0.808-1.060) | 0.262 | 0.914(0.787-1.060) | 0.233 |
|  | rs12713956 | 1.062(0.851-1.324) | 0.595 | 0.973(0.758-1.249) | 0.830 |  | 1.016(0.816-1.264) | 0.89 | 1.013(0.796-1.288) | 0.917 |

APOA-I, apolipoprotein A-I; APOB, apolipoprotein B; HOMA-IR, homeostasis model assessment for insulin resistance; MetS, metabolic syndrome.

* adjust for age, gender, BMI

Table S4. Linear regression of APOA GRS with clinical characteristics

| Characteristics | ß | P | ß* | P* |
| --- | --- | --- | --- | --- |
| BMI, Kg/m2 | -0.025 | 0.12 |  |  |
| NC, cm | -0.024 | 0.133 | -0.013 | 0.581 |
| WC, cm | -0.018 | 0.249 | -0.006 | 0.827 |
| HC, cm | -0.026 | 0.097 | -0.024 | 0.333 |
| WHR | -0.003 | 0.83 | 0.007 | 0.706 |
| Glucose (mmol/L) | -0.01 | 0.519 | -0.009 | 0.58 |
| Insulin (uU/ml) | -0.043 | **0.007** | -0.04 | **0.023** |
| HOMA-IR | -0.04 | **0.013** | -0.038 | **0.032** |
| SBP, mmHg | 0.004 | 0.789 | 0.007 | 0.68 |
| DBP, mmHg | -0.019 | 0.241 | -0.017 | 0.306 |
| TC | -0.022 | 0.164 | -0.02 | 0.213 |
| TG | -0.115 | **<0.001** | -0.115 | **<0.001** |
| HDL-C | 0.091 | **<0.001** | 0.092 | **<0.001** |
| LDL-C | 0.042 | **0.008** | 0.046 | **0.004** |
| APOA-I | 0.055 | **0.001** | 0.053 | **0.001** |
| APOB | -0.03 | 0.061 | -0.027 | 0.097 |
| APOB/APOA-I | -0.057 | **<0.001** | -0.054 | **<0.001** |
| AHI | -0.012 | 0.466 | 0.009 | 0.596 |
| Minimum SaO2 | -0.001 | 0.933 | -0.011 | 0.52 |
| ODI | -0.008 | 0.623 | 0.002 | 0.896 |
| MAI | -0.022 | 0.186 | -0.015 | 0.387 |
| ESS | 0.004 | 0.826 | 0.05 | 0.767 |

GRS: genetic risk score; BMI, body mass index; NC, neck circumference; WC, waist circumference; HC, hip circumference; WHR, waist/hip ratio; HOMA-IR, homeostasis model assessment for insulin resistance; SBP, systolic blood pressure; DBP, diastolic blood pressure; TC, total cholesterol; TG, triglyceride; HDL-C, high-density lipoprotein cholesterol; LDL-C, low-density lipoprotein; cholesterol; APOA-I, apolipoprotein A-I; APOB, apolipoprotein B; ESS, Epworth Sleepiness Scale; AHI, apnoea–hypopnea index; SaO2, oxygen saturation; ODI, oxygen desaturation index; MAI, micro-arousal index.

* adjust for age, gender, BMI

Table S5. Linear regression of APOB GRS with clinical characteristics

|  | ß | P | ß* | P* |
| --- | --- | --- | --- | --- |
| BMI, Kg/m2 | -0.009 | 0.571 |  |  |
| NC, cm | -0.017 | 0.3 | -0.018 | 0.454 |
| WC, cm | 0. 14 | 0.375 | 0.058 | 0.048 |
| HC, cm | 0.011 | 0.509 | 0.04 | 0.115 |
| WHR | 0.012 | 0.441 | 0.017 | 0.387 |
| Glucose (mmol/L) | 0.004 | 0.789 | 0.004 | 0.803 |
| Insulin (uU/ml) | 0.005 | 0.735 | 0.01 | 0.561 |
| HOMA-IR | 0 | 0.995 | 0.003 | 0.863 |
| SBP, mmHg | -0.005 | 0.75 | -0.003 | 0.875 |
| DBP, mmHg | 0.004 | 0.822 | 0.009 | 0.585 |
| TC | 0.056 | **<0.001** | 0.06 | **<0.001** |
| TG | 0.017 | 0.271 | 0.021 | 0.191 |
| HDL-C | -0.008 | 0.602 | -0.014 | 0.404 |
| LDL-C | 0.071 | **<0.001** | 0.074 | **<0.001** |
| APOA-I | -0.016 | 0.318 | -0.02 | 0.228 |
| APOB | 0.09 | **<0.001** | 0.096 | **<0.001** |
| APOB/APOA-I | 0.081 | **<0.001** | 0.089 | **<0.001** |
| AHI | 0.011 | 0.477 | 0.018 | 0.313 |
| Minimum SaO2 | -0.001 | 0.935 | -0.005 | 0.754 |
| ODI | 0.009 | 0.58 | 0.018 | 0.335 |
| MAI | -0.002 | 0.886 | 0 | 1 |
| ESS | 0.004 | 0.832 | 0.001 | 0.974 |

GRS: genetic risk score; BMI, body mass index; NC, neck circumference; WC, waist circumference; HC, hip circumference; WHR, waist/hip ratio; HOMA-IR, homeostasis model assessment for insulin resistance; SBP, systolic blood pressure; DBP, diastolic blood pressure; TC, total cholesterol; TG, triglyceride; HDL-C, high-density lipoprotein cholesterol; LDL-C, low-density lipoprotein; cholesterol; APOA-I, apolipoprotein A-I; APOB, apolipoprotein B; ESS, Epworth Sleepiness Scale; AHI, apnoea–hypopnea index; SaO2, oxygen saturation; ODI, oxygen desaturation index; MAI, micro-arousal index.

* adjust for age, gender, BMI

Table S6. The stepwise multivariate linear regression model for predicting HOMA-IR.

|  | ß | R^2^ | P |
| --- | --- | --- | --- |
| **Model 1** |  |  |  |
| APOA GRS | -0.031 | 0.00099 | 0.031 |
| Age | - | - | - |
| Gender | 0.038 | 0.0014 | 0.009 |
| BMI | 0.423 | 0.180 | <0.001 |
| **Model 2** |  |  |  |
| APOA GRS | -0.032 | 0.0010 | 0.028 |
| Age | - | - | - |
| Gender | 0.038 | 0.0014 | 0.009 |
| BMI | 0.374 | 0.180 | <0.001 |
| AHI | 0.109 | 0.0094 | <0.001 |

APOA-I, apolipoprotein A-I; APOB, apolipoprotein B; GRS: genetic risk score; BMI, body mass index; AHI, apnoea–hypopnea index.
